# Unmet Communication and Information Needs for Patients with IBD: Implications for Mobile Health Technology

**DOI:** 10.9734/BJMMR/2016/21884

**Published:** 2016

**Authors:** Sameer Khan, Florence Dasrath, Sara Farghaly, Emamuzo Otobo, Muhammad Safwan Riaz, Jason Rogers, Anabella Castillo, Ashish Atreja, Health PROMISE Consortium Group

**Affiliations:** 1 Icahn School of Medicine at Mount Sinai, New York, USA

**Keywords:** IBD, SIBDQ, mHealth, engagement

## Abstract

**Aims:**

In order to develop an application that addresses the most significant challenges facing IBD patients, this qualitative study explored the major hurdles of living with IBD, the information needs of IBD patients, and how application technology may be used to improve quality of life.

**Methods:**

15 IBD patients participated in two focus groups of 120 minutes each. Data collection was achieved by combining focus groups with surveys and direct observation of patients looking at a patient-engaged app (HealthPROMISE) screenshots. The survey elicited information on demographics, health literacy and quality of life through the Short IBD Questionnaire (SIBDQ).

**Results:**

The needs of IBD patients center around communication as it relates to both patient information needs and navigating the social impacts of IBD on patients’ lives:

- Communication Challenges regarding Information Needs: Patients cited a doctor-patient communication divide where there is a continued lack of goal setting when discussing treatments and a lack of objectivity in disease control. When objectively compared with the SIBDQ, nearly half of the patients in the focus groups wrongly estimated their IBD control.
- Communication Challenges regarding Social Impacts of IBD: Patients strongly felt that while IBD disrupts routines, adds significant stress, and contributes to a sense of isolation, the impact of these issues would be significantly alleviated through more conversation and better support.
- Implication for Mobile Health Solutions: Patients want a tool that improves tracking of symptoms, medication adherence and provides education. Physician feedback to patient input on an application is required for long-term sustainability.

**Conclusions:**

IBD patients need mobile health technologies that evaluate disease control and the goals of care. Patients feel an objective assessment of their disease control, goal setting and physician feedback will greatly enhance utilization of all mobile health applications.

## 1. INTRODUCTION

Inflammatory Bowel Disease (IBD) is a chronic condition of the bowel that affects over 1 million people in the United States [1]. Although the incidence of IBD is rising, the precise cause of the disease remains unknown. The two major subtypes of Inflammatory Bowel Disease are Crohn’s Disease and Ulcerative Colitis. In Crohn’s, inflammation may occur in any part of the gastrointestinal tract ranging from the mouth to the anus. Moreover, Crohn’s affects all layers of the intestinal wall [1]. In contrast, in Ulcerative Colitis inflammation originates in the rectum and may spread across the entire length of the colon [1].

Medical treatment for IBD has improved significantly in recent years, however, current efforts are largely ameliorative rather than curative. As a result, IBD patients have to cope with a lifelong condition in which there are commonly remissions and relapses. This quality makes IBD patients the ideal candidates to target for improved self-management when it comes to care. Recent studies suggest that most IBD patients still feel insufficiently informed and want greater involvement in their treatment [2]. Patients want information that helps them take care of themselves more actively [3].

Facilitating patient-engaged care does more than just empower patients; it also leads to better outcomes. IBD patients who participate in their own care and share in decision-making have appreciably improved outcomes when compared to patients who do not [4]. For healthcare teams, the lingering question remains – how do we better engage patients without placing increased time constraints on healthcare staff? The answer, in part, lies in new technologies.

In the age of the Smartphone and the endless barrage of information on the web, the demand for healthcare that optimizes on instant communication and improved information delivery is growing. Mobile phone usage is highest among historically difficult to access populations including adolescents, young adults, low-income populations, less educated adults, and those with less stable home addresses [5]. Furthermore, there is significant support in the medical community that mobile / internet technologies can reduce the burden of disease by enabling improved patient involvement. Such advances have already been shown to be successful and well received by patients with chronic diseases other than IBD as well [6].

As the Internet sources providing information to patients multiplies, there is growing concern about the quality of the information and the difficulties in finding answers to specific questions [7]. Easily accessible mobile health applications with content created by healthcare professionals may be the best way to address these troubling issues.

The Mount Sinai Inflammatory Bowel Disease (IBD) Center, a tertiary care center, has received funding to develop a patient-centric application that addresses the most significant challenges encumbering patients with IBD today. Creating an application that properly engages a patient population is an enormous challenge. Often mobile health apps and web-based programs suffer from poor utilization rates with adherence rates as low as 5 percent [8,9]. Abysmal usage nullifies any practical benefit of an application.

In order to design an effective application and better understand the needs of IBD patients, we conducted a qualitative study using focus groups. Originally, this study was designed to explore the thoughts of IBD patients regarding: 1) The major hurdles of living with IBD 2) The information needs of patients with Crohn’s or Ulcerative Colitis and 3) How application technology can be used to improve patient quality of life.

## 2. METHODS

Focus group methodology [10] was used to engage IBD patients and discuss health concerns, major information needs, and the best way that mobile technology can help address these needs. We used multiple methods of data collection commonly used in studies of human computer interactions, combining focus groups with surveys and direct observation of patients looking at screenshots from a patient-engaged application (HealthPROMISE). http://healthpromise.org/

### 2.1 Subject and Focus Group Procedure

2 focus groups (lasting from 90 to 120 minutes) were conducted with 15 patients from the tri-state area (NY, NJ, CT). Patients were selected from the Mount Sinai Crohn’s and Colitis Registry (MSCCR). Patient demographics and informed consent were collected from all participating parties of this study (Table 1). Assurance of confidentiality was given to all participants. The facilitators for both focus groups were kept constant to ensure consistency.

Prior to beginning, patients were given a short survey including demographic information, computer literacy, and the Short IBD Questionnaire (SIBDQ). The SIBDQ is used to evaluate a patient’s quality of life in a consistent and standardized fashion. The quality of life is a measure of the impact of a chronic illness on a patient’s function, behavior or performance. However, it also reflects patient perceptions, beliefs and attitudes [11]. The SIBDQ was administered in order to gain insight into patients’ perception of how they are doing in comparison to a standardize metric that evaluates how well controlled their IBD is.

The focus groups largely began with general questions: “What is the major challenge you face as a patient with IBD?” Discussion and follow-up questions were used to encourage patients to elaborate with as much detail as possible, but also to generate consensus around shared concerns. As the focus group continued additional topics were also explored including health information needs and technology platforms that could meet these needs. Only two focus groups were required to reach saturation.

#### 2.1.1 Data analysis

Qualitative inductive analysis was used. All focus groups were recorded and transcribed by a team including the first, second, and third authors. Once transcribed, the focus group discussion was read to develop a general understanding of the overall thoughts and concerns of patients. Direct quotes and other phrases relevant to the aim of the research question were identified and coded. Similar codes were then categorized to develop main themes. In this process, the research group worked individually to prevent influence on their fellow researchers’ analysis of the text. The analyses were then compared and consensus reached. The most important needs and social impacts were determined by the number of mentions by different patients and the number of coded single-line text units.

## 3. RESULTS

### 3.1 IBD Patient Needs

Prior to the focus groups, we anticipated learning about the information deficits that, if met, would empower patients to better manage their IBD. However, in both focus groups, it rapidly became evident that the needs of IBD patients center more around *communication* as it relates to both information needs and the social impact of IBD on patients’ lives. These results are summarized in Table 2.

#### 3.1.1 Communication challenges regarding information needs

Many patients expressed frustration stemming from ineffectual communication with members of the medical community. A clear majority of patients agreed that there is a *doctor-patient communication divide*. Physicians often do not clearly articulate to patients what they have and the full ramifications of their diagnosis. Explanations of treatment approaches are frequently clinical and thus, obscure patient understanding. However, doctors and patients are jointly responsible for this communication void. Patients, due to a myriad of factors such as: discomfort in discussing the breadth of their symptoms, nervousness, and the desire to “look okay”, often minimize the extent of their disease in relating it to their doctor. This failure to inform the physician of the wide variety of symptoms deepens the rift in communication, leading to a poorer standard of care and frustration on the side of the patient.

One of the most surprising themes brought up by patients was *lack of goal setting* between patients and physicians when discussing the objective of treatment. Patients repeatedly voiced exasperation with the unclear long-term goals set by their physician. In numerous cases, long-term goals were never even discussed!

Together, the doctor-patient communication divide and the frequent lack of goal setting culminate to create a vacuum, which stifles information exchange. In response to this uncertainty, patients frequently turn to *alternative online resources*, which often provide counterfactual and sometimes harmful information if they choose to follow it.

#### 3.1.2 Communication challenges regarding social impacts of IBD

It is well known that there are significant social repercussions for patients with IBD. In discussions with the patients however, it became clear that above all else, *communication* about these social impacts is paramount. As one patient put it, “even this focus group is therapeutic!” IBD patients yearn for a setting where they can share experiences about social setbacks and learn about strategies to better handle them.

Patients highlighted the following social challenges as the ones that most commonly affect IBD patients and which require more conversation and better support: 1) *IBD disrupts routine,* 2) *IBD patients cope with stress and* 3) *IBD patients deal with a sense of isolation*.

#### 3.1.3 How is my IBD? Patients do not know

**Fig. 1:**
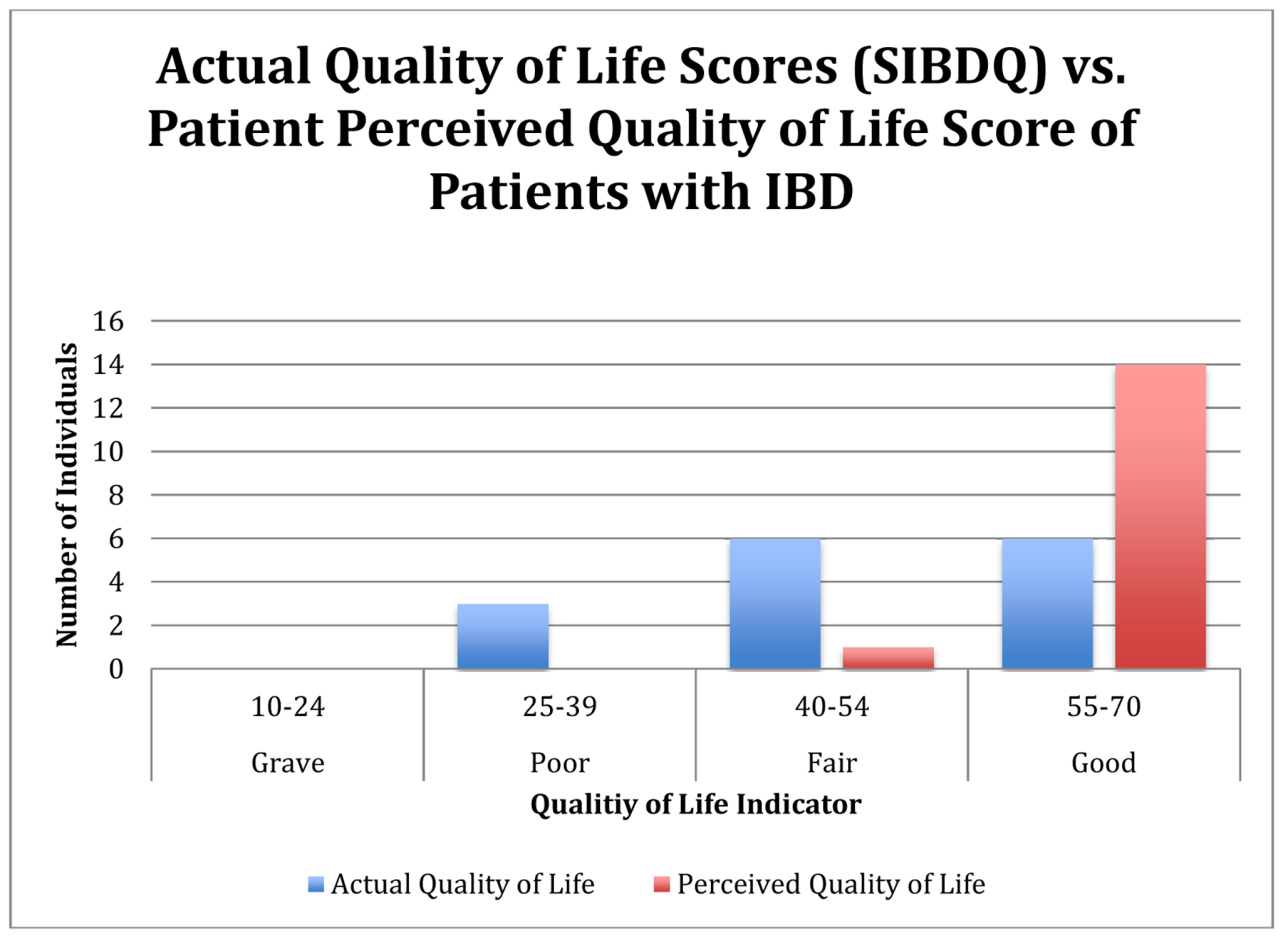
Perceived vs. actual quality of life

Patients with IBD do not accurately know the control of their disease. In the focus groups, patients were asked about their own perception regarding the control of their IBD as compared to their quality of life (QOL) score based on the standardized metric of SIBDQ. Four intervals were created to categorize different scores. 10–24 described a grave condition for patients, 25–39 described poor control, 40–54 described fair control and 55–70 described good control. After results between perceived and actual QOL were compared, it was found *patients misperceive their IBD control*. As can be seen in Fig. 1, 14 patients perceived they were in “good” control and 1 patient in “fair” control. However, according to the SIBDQ, 6 patients were in “good” control, 6 patients were in “fair “control and, although none of the patients believed they were in “poor” control, 3 of them were. In summary, 9 out of 15 patients (60%) wrongly estimated their IBD control.

There is a major discrepancy between how patients understand their disease status as compared to their actual status as measured by a standardized tool.

### 3.2 Mobile Technology Solutions

The last portion of the focus group centered on how mobile health technologies can successfully address some of the major needs of IBD patients. Although this discussion generated a wide range of responses, a consensus was reached about three main items. These are highlighted with quotes from the focus group in Table 3.

Patients felt that an application must be able to inform their medical team of their health status and how they are doing in the interval of time between visits. Any application would have to enable improved *surveillance of symptoms and medication adherence*. This would fill a troubling gap in their doctor’s understanding of a patient’s status when not in the doctor’s office. Furthermore, an application that tracks a patient’s medications would enable better self-management.

While interactions with one’s physician are essential, patients also elaborated on the need for an IBD community where patients can share experiences, receive support and learn from one another. Ideally, healthcare professionals would provide input in this *living community* according to their expertise providing much needed credibility to the source.

Finally, when exploring the specific topic of what would be the most essential feature for a health application to be successful, all patients agreed that their doctor needed to keep up with any new information they were providing otherwise the effort is not worth it. *Physician involvement and feedback* is crucial as a form of positive reinforcement for patients to keep using the application.

## 4. DISCUSSION

As IBD has gained prominence, the medical community has learned about the impact of IBD on the lives of patients and the need for better patient education [12]. This realization has occurred amidst significant new developments in technology and its use. Presently, 75% of the US population has access to the Internet, a percentage that has steadily increased the past 20 years [13, 14]. In light of this development, there is a growing potential for eHealth technologies to address patient needs that are commonly overlooked by traditional systems of care. In this study, social impacts and needs were explored with the intention of divining how mobile health technology may help alleviate patient concerns.

Our study revealed that the core needs of IBD patients revolve around, and are heavily dependent upon, improved communication between physicians and patients as well as amongst patients themselves. As previous studies have established and as patients in our focus groups suggested, inadequate access to healthcare professionals and a lack of time to discuss all issues are major constraints on existing communication frameworks [6,7]. Considering these hindrances, technology solutions are the most appropriate.

### 4.1 IBD Application Answers for Information Needs

**Fig. 2:**
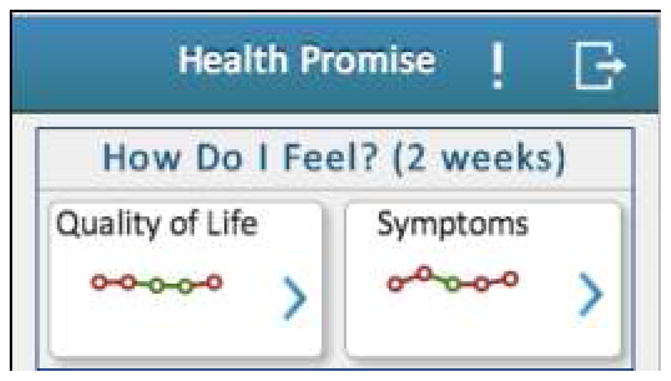
Quality of life trackers

Among specific information needs, more than half of the patients do not know their quality of life and wrongly estimated the control of their IBD. In a European study, Mitchell et al. [15] reported that in 50% of cases QOL is not even discussed by either patient or physician. Frequently, this is because QOL is not formally evaluated. To address this major issue, a mobile health application should regularly report a patient’s QOL score to both the physician and patient. This feature would reduce time spent capturing the QOL during an office visit and encourage a more focused conversation. To address this need in our own application, HealthPROMISE, QOL scores are reported every two weeks in a longitudinal manner that allows long-term tracking as seen in Fig. 2.

Additional communication challenges regarding information needs outlined by patients included:

1. Doctors do not properly explain the lifelong implications of IBD [11].
2. Patients seek alternative sources of information online that are unreliable.
3. Patients regularly fail to elaborate on the full expanse of their symptoms.
4. Treatments and goals of treatments are scantily discussed.

Schlomerich et al. [16] reported in a 1983 study that 77% of patients with IBD felt insufficiently informed about their disease and would like more information. In part, the solution to this problem identified by the patient in this study in items 1 and 2 above may lie in offering education to IBD patients as part of a mobile health application. Previous studies suggest that patient education improves patient satisfaction [17]. This could include physician-led videos explaining IBD conventional treatments as well as potential alternatives. As per a recommendation from the focus groups, an education section could also connect patients to recent literature in IBD. While the best format to integrate patient education into a mobile health application is still being refined for HealthPROMISE, the patient desire and need for more information is clear.

**Fig. 3:**
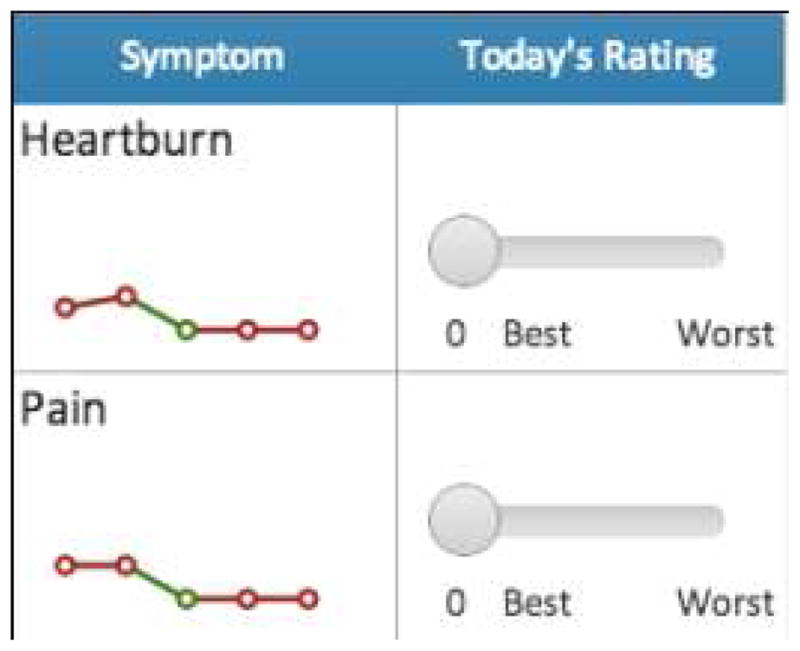
Symptoms

Concerning item 3, expecting a physician to elicit, or a patient to recall, all relevant recent symptoms are a difficult challenge. However, mobile health applications are ideal for circumventing such challenges around ideal practices due to time limitations. An application can document the majority of symptoms an IBD patient is experiencing and report them to physicians, thereby facilitating a deeper understanding of a patient’s condition. Creating a venue for information sharing about symptoms may prompt conversations and initiate modifications in treatment that allow physicians to address problems in a more expedient manner. A potential system for chronicling symptoms in response to patient feedback that is being used for HealthPROMISE may be observed in Fig. 3.

**Fig. 4:**
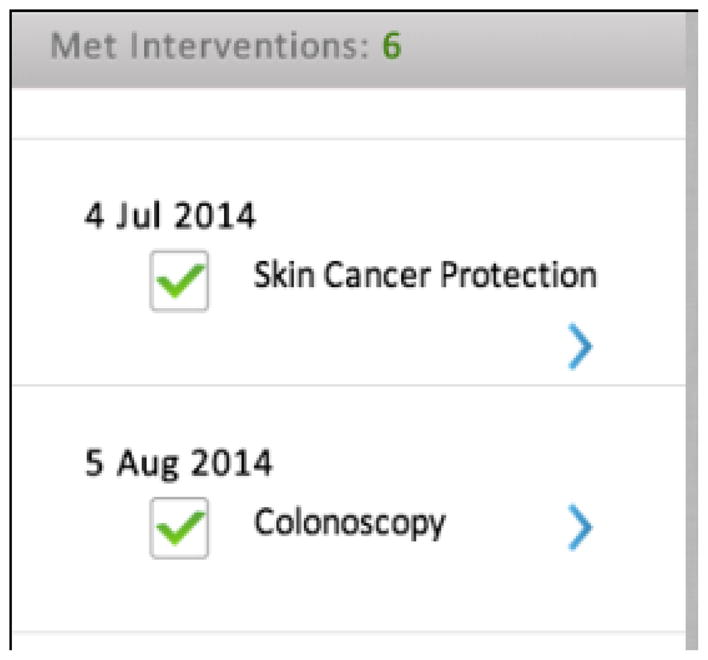
Monitoring quality indicators

Baars et al. [4] in a study with 1093 IBD patients found that 98% felt it was important to be involved in the decision-making process regarding medical treatment options and goals. In item 4, as patients in the focus group highlighted, goals are only inconsistently discussed if at all. While standardizing the goals of treatment may be difficult, the American Gastroenterological Association (AGA) has generated key IBD quality indicator measures that are important to meet for all physicians treating IBD. In HealthPROMISE, the major quality indicators tracked by gastroenterologists treating IBD are included for patients to monitor, update and discuss with their physician (Fig. 4). This may elucidate for patients the rationale behind certain treatments and encourage conversations between patients and physicians on this topic.

### 4.2 IBD Application Answers for Social Impacts

In our study, it rapidly became evident that IBD has an enormous impact on the ability of patients to have relationships and manage normal activities [18]. Through the course of the focus groups, consensus readily formed around: 1) that IBD disrupts routines, 2) IBD patients cope with stress and 3) patients deal with a sense of isolation.

For all of these social challenges, patients spoke of a desperate need for more conversation about said topics with physicians, but also fellow patients. In HealthPROMISE, the QOL score [18] and the tracking of symptoms that include anxiety and depression may in part aid in discerning whether a patient is suffering from depression or mental stress. Once such patients are identified, the appropriate psychological interventions may be pursued. However, the key to addressing the need for more conversation may actually rest in a proposition made by patients from the focus group, that is, a “living physician-patient community” online. Patients described a setting where they can share experiences, discuss treatments, and support and learn from one another. We hope to pursue this in a second version of Health PROMISE.

Psychological eHealth interventions are associated with meaningful improvements in QOL and the reduction of patient symptoms [19]. This can include skills-based training, cognitive behavior therapy, or expressive writing [19]. Ultimately, there is great potential for integration with mobile health applications or technology.

### 4.3 Ensuring Patient Engagement with Mobile Health Technology

The success of any application is heavily dependent on utilization. Our study revealed that for a mobile health application, such as HealthPROMISE, to succeed, physician involvement and feedback is vital. Patients cannot be asked to regularly provide novel information about their symptoms or QOL if physicians do not clearly demonstrate that they are receiving this information and acting on it.

To maintain physician engagement with HealthPROMISE, there is a physician panel that updates clinicians on new information a patient has submitted with periodic reminders to contact the patient to provide feedback on recent submissions (Physician panel pictured below).

## 5. CONCLUSION

This study underscores that many of the difficulties that IBD patients face in terms of information needs and handling the social impacts of IBD are the product of communication challenges. Naturally, mobile health technologies lend themselves to networking and rapid communication that may be ideal for overcoming many patient needs. However, the success of any eHealth application requires adequate patient utilization, which itself, is dependent on physician engagement.

## Figures and Tables

**Fig. 5:**
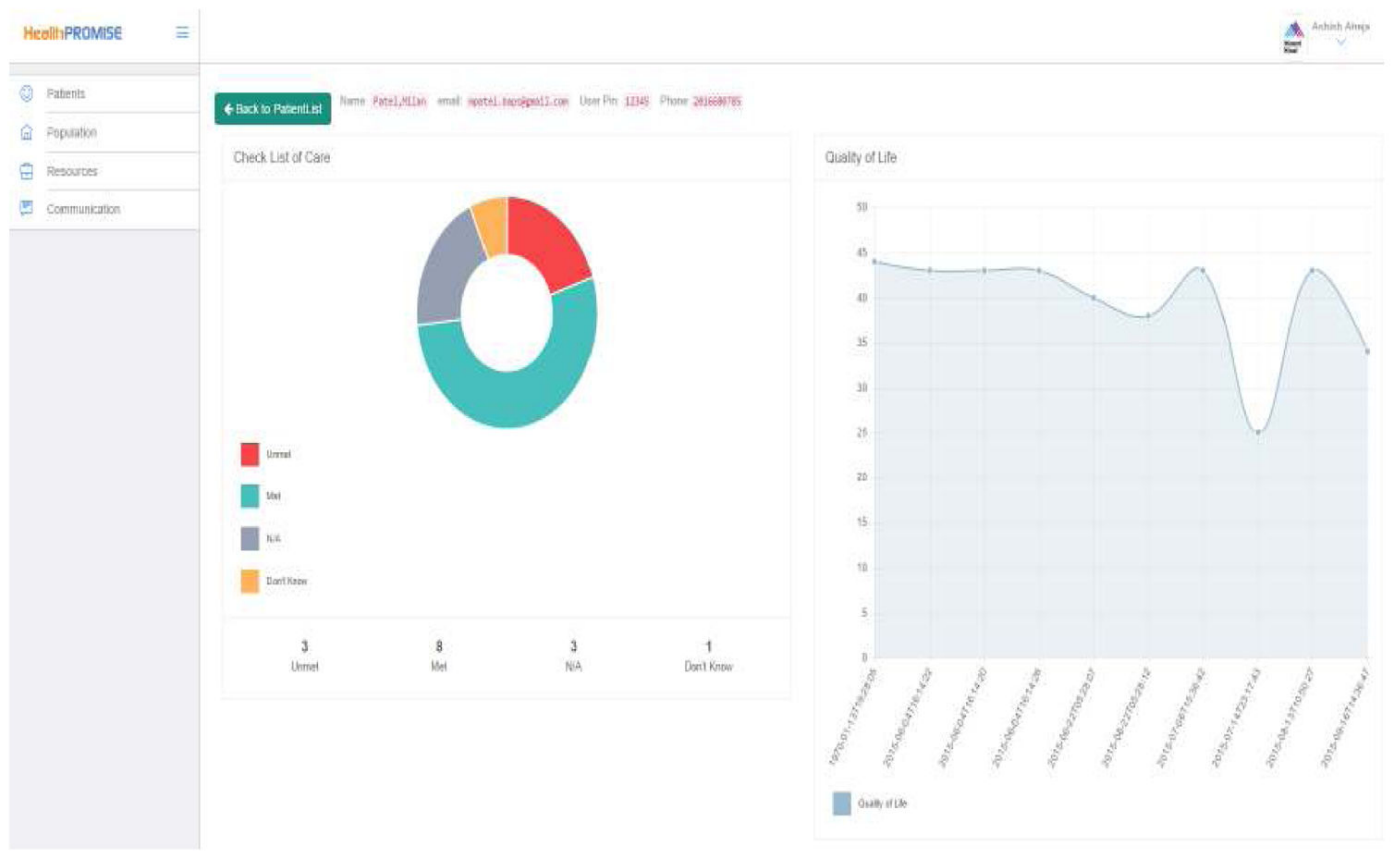
Physician panel to facilitate patient follow-up

**Table 1:** Background data of the participants

Variable	
English as the primary language	15(100%)
**Race/Ethnicity**	
Caucasian	8(53.3%)
Black/African American	4(26.6%)
Hispanic	3(20%)
**Gender**	
Male	5(33.3%)
Female	10(66.7%)
**Age**	
18–25	4(26.6%)
26–35	4(26.6%)
36–45	2(13.3%)
46+	5(33.3%)
**Education completed**	
High School	2(13.3%)
1–3 years of college	3(20%)
4+ years of college	10(66.7%)
**Total annual income of patient and spouse**	
$10,001–$20,000	3(21.4%)
$20,001–$35,000	2(14.3%)
$35,001–$50,000	2(14.3%)
$50,001–$100,000	2(14.3%)
$100,001 or more	5(35.7%)
Computer access at home or work	14(93.3%)
Computer proficient	14(93.3%)
Owns a smartphone or tablet	14(93.3%)
**App usage on smartphone or tablet**	
Everyday	10(66.7%)
Every 2–3 days	2(13.3%)
Once a week	1(6.7%)
Less than once a week	1(6.7%)
Never	1(6.7%)

**Table 2:** Patient needs

Patient need	Quote(s)
**a) Communication challenges regarding information needs**	
*Doctor-Patient Communication Divide* Patients feel that doctors do not understand what they are experiencing. Doctors tend to dismiss to patient-offered therapeutic advice.	“I act like everything is okay”. “Felt like a guinea pig, one medication after the next”. “Doctors medicate the problem, but are not treating the problem”. “Doctors say cold word and you don’t understand what is being said”. “Every time he says he asks how are you doing and I say I’m good. That’s all I say”.
*Lack of Goal Setting* Patients do not have the opportunity to discuss the goals of their treatment plan with their physician or encounter disparities between the goals set.	“I never talk about goals with my doctor”. “You have to drive the conversation over there. It would be more human if that were part of the plan”. “There aren’t clear enough goals…. I’d like to know the strides that are being made”.
*Alternative Sources of Information* As a result of inadequate information concerning health status and treatment from professionals, patients independently search for alternatives online, which are often unreliable.	“I know someone who drinks a teaspoon of peroxide and water everyday and she said she doesn’t have problems with her colitis”. “Reddit has a sub community that people talk about their lifestyle”. “ I am scared using information online. It’s not verified, but it fills the gap for a network I need”.
**b) Communication challenges regarding social impacts of IBD**	
*IBD disrupts routines* Patients-- unable to commit to tasks that require regular and continuous involvement because of the unpredictable nature of their IBD-- want to have an IBD community that helps them make progress.	“My IBD makes it difficult to maintain a job”. “I wonder if I could survive with a job”. “I struggle with regular exercise and to do what I want with my life”.
*Stress* IBD and its symptoms often cause stress, drastically affecting the quality of life of patients. Often, they feel there is not anyone to speak with them about stress.	“Coping with stress is a challenge. Trying to be stress free is so important makes me feel better, but sometimes its inevitable”. “Stress is causing me to lose my hair”.
*Sense of Isolation/Inability to communicate about disease* IBD patients find it hard to explain their situation to people when asked. Patients do not want family to be extensively involved, so they tend to minimize their symptoms. They need other patients to talk to about living with disease.	“I don’t discuss IBD with anyone. My husband is always asking me if I’m okay. I act like everything is okay…” “My kids don’t know anything. All they know is that I went to the bathroom…” “Explaining it to people. How you explain it to others”. “It happening is a major challenge and explaining it to people. How you explain it to others”.

**Table 3:** Mobile health tech. recommendations

Recommendation for role of technology	Quote(s)
*Better surveillance of symptoms and medication adherence between clinical visits* Doctors are often unaware the symptoms that patients experience between clinic visits as well as how well they are adhering to their treatment regimen.	“…. I took liberties and changed the dosages on my own.” “Is it possible to have a reminder when my medications are running low?”
*Living physician-patient community* No popular community exists for patients to discuss their experiences and treatment advice for other patients with a physician present to moderate the advice and its validity.	“Focus groups are helpful. This is where you get information and network with other people that have the disease…. We can benefit from each other’s information and experiences.”
*Physician engagement is necessary* Doctors need to be up to date with changes patients are making in an application so that new information can have an impact on care and improve patient-doctor communication.	“Knowing that it is connected to the doctor.” “It is essential to know that the doctor is connected to the application.” “Physicians have to adopt or I lose faith.”

## References

[1] Auria JPD, Kelly M (2013). Inflammatory Bowel Disease: Top resources for children, adolescents, and their families. Journal of Pediatric Health Care.

[2] Kennedy AP, Nelson E, Reeves D, Richardson G, Roberts C, Robinson A, Rogers AE, Sculpher M, Thompson DG (2004). A randomised controlled trial to assess the effectiveness and cost of a patient orientated self-management approach to chronic inflammatory bowel disease. Gut.

[3] Lesnovska KP, Börjeson S, Hjortswang H, Frisman GH (2014). What do patients need to know? Living with inflammatory bowel disease. J Clin Nurs.

[4] Baars JE, Markus T, Kuipers EJ, van der Woude CJ (2010). Patients’ preferences regarding shared decision-making in the treatment of inflammatory bowel disease: Results from a patient-empowerment study. Digestion.

[5] Sharifi M, Dryden EM, Horan CM, Price S, Marshall R, Hacker K, Finkelstein JA, Taveras EM (2013). Leveraging text messaging and mobile technology to support pediatric obesity-related behavior change: A qualitative study using parent focus groups and interviews. J Med Internet Res.

[6] Landy J, Peake ST, Akbar A, Hart AL (2012). BSG information group symposium & free papers: Social media and apps: New opportunities, new risks: OC-160 Telemedicine systems in IBD management-are patients ready?. Gut.

[7] Skinner H, Biscope S, Poland B, Goldberg E (2003). How adolescents use technology for health information: implications for health professionals from focus group studies. J Med Internet Res.

[8] Mañanes G, Vallejo MA (2014). Usage and effectiveness of a fully automated, open-access, Spanish Web-based smoking cessation program: Randomized controlled trial. J Med Internet Res.

[9] Helander E, Kaipainen K, Korhonen I, Wansink B (2014). Factors related to sustained use of a free mobile app for dietary self-monitoring with photography and peer feedback: Retrospective cohort study. J Med Internet Res.

[10] Morgan David L (1993). Successful focus groups: Advancing the State of the Art (SAGE Focus Editions).

[11] Irvine EJ (2004). Review article: Patients’ fears and unmet needs in inflammatory bowel disease. Aliment Pharmacol Ther.

[12] Ghosh S, Mitchell R (2007). Impact of inflammatory bowel disease on quality of life: Results of the European Federation of Crohn’s and Ulcerative Colitis Associations (EFCCA) patient survey. J Crohns Colitis.

[13] United States Census Bureau http://www.census.gov/hhes/computer/.

[14] (2003). eHealth’s Influence Continues to Grow as Usage of the Internet by Physicians and Patients Increases. Health care news.

[15] Mitchell R, Kremer A, Westwood N, Younge L, Ghosh S (2009). Talking about life and IBD: A paradigm for improving patient-physician communication. J Crohns Colitis.

[16] Scholmerich J, Sedlak P, Hoppe-Seyler P, Gerok W (1987). The information needs and fears of patients with inflammatory bowel disease. Hepatogastroenterology.

[17] Waters BM, Jensen L, Fedorak RN (2005). Effects of formal education for patients with inflammatory bowel disease: A randomized control trial. Can J Gastroenterol.

[18] Agostini A, Moretti M, Calabrese C, Rizzello F, Gionchetti P, Ercolani M, Campieri M (2014). Attachment and quality of life in patients with inflammatory bowel disease. Int J Colorectal Dis.

[19] Knowles SR, Mikocka-Walus A (2014). Utilization and efficacy of internet-based eHealth technology in gastroenterology: A systematic review. Scand J Gastroenterol.

